# Alpha‐fetoprotein accelerates the progression of hepatocellular carcinoma by promoting *Bcl‐2* gene expression through an RA‐RAR signalling pathway

**DOI:** 10.1111/jcmm.15962

**Published:** 2020-10-22

**Authors:** Chao Zhang, Jiangtao Zhang, Jing Wang, Ying Yan, Chuanbao Zhang

**Affiliations:** ^1^ National Center for Clinical Laboratories National Center of Gerontology Beijing Hospital Beijing China; ^2^ Institute of Geriatric Medicine Chinese Academy of Medical Sciences Beijing Hospital Beijing China

**Keywords:** alpha‐fetoprotein, Bcl‐2, hepatocellular carcinoma, RA‐RAR signalling pathway

## Abstract

Previous studies have found that alpha‐fetoprotein (AFP) can promote the proliferation of hepatoma cells and accelerate the progression of hepatocellular carcinoma (HCC). However, the exact mechanism of action remains unclear. Recent bioinformatics studies have predicted the possible interaction between AFP and retinoic acid receptors (RARs). Thus, the purpose of this study was to investigate the molecular mechanism through which AFP promotes tumour cell proliferation by interfering with the RA‐RAR signal pathway. Our data indicated that AFP could significantly promote the proliferation and weaken ATRA‐induced apoptosis of hepatoma cells. Besides, cytoplasmic AFP interacts with RAR, disrupting its entrance into the nucleus, which in turn affects the expression of the *Bcl‐2* gene. In addition, knockdown of AFP in HepG2 cells was synchronously associated with an incremental increase of RAR binding to DNA, as well as down‐regulation of Bcl‐2; the opposite effect was observed in AFP gene‐transfected HLE cells. Moreover, a similar effect of AFP was detected in tumour tissues with high serum AFP, but not in adjacent non‐cancerous liver tissues, or HCC tissues with low serum AFP levels. These results indicate that AFP acts as signalling molecule and prevents RAR from entering into the nucleus by interacting with RAR, thereby promoting the expression of Bcl‐2. Our data reveal a novel mechanism through which AFP regulates Bcl‐2 expression and further suggest that AFP may be used as a novel target for treating HCC.

## 1 INTRODUCTION

Alpha‐fetoprotein (AFP) is an embryonic‐specific alpha‐globulin produced by the foetal liver or yolk sac, and an important component of mammalian serum in the early embryonic stage.^1^, ^2^, ^3^ Levels of AFP in foetal serum drop rapidly after birth and are almost undetectable in adult stage AFP.^4^ Liver damage and a certain tumour can significantly increase AFP concentrations.

Alpha‐fetoprotein has been widely applied as a tumour biomarker in detecting primary hepatocellular carcinoma (HCC), with high specificity.^5^ Previous studies have reported that 70%‐80% of patients with primary hepatocellular carcinoma have elevated serum AFP levels.^6^ Besides, existing studies have also confirmed that AFP can promote the proliferation of tumour cells and inhibit the apoptosis induced by anti‐cancer drugs. Even in AFP‐negative hepatoma cells, exogenous AFP administration can significantly increase the proliferation of cancer cells.^7^, ^8^, ^9^ Yet, the exact action mechanism of AFP in promoting proliferation and inhibiting apoptosis remains unclear.

Mizejewski et al analysed the molecular structure and amino acid sequence of AFP using bioinformatics, revealing that some amino acid sequences in the third structural domain of human AFP protein have transcriptional regulation functions.^10^, ^11^, ^12^, ^13^ This particular sequence is characterized by eight to nine hydrophobic amino acid repeats (heptads), forming a leucine‐zipper dimerization motif, which is a classical domain combined with hormone response elements. The molecular characteristics of this dimerization motif on AFP are also present in the amino acid sequences of members of the steroid/thyroid receptor superfamily, that is multiple stretches of 7‐8 amino acid sequences of contiguous and non‐contiguous hydrophobic repeats or heptads, which are similar to the amino acid extension sequences of members of the thyroid/retinoic acid superfamily.^14^ At the same time, multiple amino acid sequences of full‐length AFP were analysed. The results showed that AFP could match with various nuclear transcription factors and nuclear receptor sequences. Although the most conservative heptad matching (50%‐60%) occurred between AFP and the retinoic acid receptors (RAR), both steroid and thyroid superfamily members displayed amino acid matching in 5 of 9 of the AFP predicted heptads. These structural characteristics provide guidance and research direction on mechanisms, through which AFP exert its biological function in cells.

Bcl‐2 gene is a proto‐oncogene and an essential member of the anti‐apoptotic family. Previous studies have shown that inhibition of Bcl‐2 expression can enhance the sensitivity of hepatoma cells to anti‐tumour drugs.^15^, ^16^ It has been suggested that ATRA can inhibit Bcl‐2 expression by activating RAR, thereby promoting tumour cell apoptosis.^17^, ^18^ RAR, a natural receptor of ATRA, is highly likely to bind to AFP. We hypothesized that AFP could interfere with the RA‐RAR signalling pathway by binding with RAR, and thereby inhibiting the transcriptional regulation of RAR in hepatoma cells.

In view of these findings, it is critical to determine whether AFP participates in reducing RAR nuclear translocations and binding to DNA via interaction with RAR, thus promoting Bcl‐2 transcription. Therefore, the aim of this study was to explore the role of cytoplasmic AFP in RAR‐mediated Bcl‐2 transcription and provide experimental support for further elucidation of the mechanisms underlying hepatocarcinogenesis.

## 2 MATERIAL AND METHODS

### 2.1 Cell culture

HepG2 (AFP‐positive) cells were purchased from ATCC; HLE (AFP‐negative) cells were a gift from the Key Laboratory of Molecular Biology, Hainan Medical College. All cells were cultured in Gibco DMEM (high glucose) medium supplemented with 10% FCS in a humidified atmosphere containing 5% CO_2_/95% air at 37°C.

### 2.2 Plasmid transfection

pcDNA3.1 (+)‐Bcl‐2 was constructed with Hind III/EcoR I. All the plasmids used in these transfection experiments were prepared using a Large‐scale Purification Kit (Tiangen Biotech), following the manufacturer's instruction. Cells were transfected with plasmid and siRNA using Lipofectamine 2000 (Thermo), according to the application guide of the product.

### 2.3 qRT‐PCR

The expression levels of AFP and Bcl‐2 mRNA were evaluated by quantitative real‐time PCR, as previously described.^1^ Relative concentrations of mRNA are presented as mean fold change of samples compared with the control. β‐actin was used as an internal reference. The primers involved in the experiment are shown in Table S1.

### 2.4 Western blot

Western blotting was used to analyse the expression of AFP, RAR and Bcl‐2 in hepatoma cell lines as described. Primary antibodies against AFP, Bcl‐2 (Proteintech), RAR and β‐actin (Wanleibio) were used according to the instructions.^1^ The secondary antibodies conjugated to horseradish peroxidase were purchased from Zhongshan Biol Tech.

### 2.5 Nuclear translocation of RAR

Laser confocal microscopy was performed to evaluate the nuclear translocation of RAR in hepatoma cell lines. Rabbit anti‐RAR antibody and secondary anti‐rabbit IgG antibodies conjugated with Alexa Fluor 488 were purchased from Proteintech Biotech and Zhongshan Boil Tech, respectively. Treated cells were viewed and captured with a Laser Confocal Microscope (Leica TCSNT SP2).

### 2.6 Coimmunoprecipitation (CoIP)

The interaction of AFP and RAR in cell lines was evaluated by CoIP assay with antibodies purchased from Proteintech and Santa Cruz Biotechnology, as previously described.^1^

### 2.7 Glutathione‐S‐transferase (GST) pull‐down assay

Glutathione‐S‐transferase (GST) pull‐down assay was used to verify the interaction between AFP and RAR. A GST‐RAR clone was purchased from Beijing FunGenome. GST pull‐down experiments were performed using the TNT T7 Quick Coupled Transcription/Translation System Kit (Promega), followed by Western blotting to evaluate the interaction between TNT system translation products and the GST‐RAR fusion proteins.

### 2.8 RNAi assay

The RNA interference technique was used to verify the effect of AFP on Bcl‐2 expression and RAR nuclear translocation, as previously described.^19^ The expression levels of AFP and Bcl‐2 mRNA/protein and the nuclear translocation of RAR were evaluated using Western blotting and qRT‐PCR after cell transfection with siRNAs for 36 hours.

### 2.9 Flow cytometry

Flow cytometric assay was performed in a standard manner to determine the effect of ATRA, AFP and Bcl‐2 on apoptosis. Apoptosis induced by ATRA was analysed using Annexin V/PI Apoptosis Detection Kit (Dojindo Laboratories) on BD FACSCantoTM Π flow cytometry instrument (BD). The apoptosis levels of cells were determined by DNA analysis.

### 2.10 Detection of the viability of hepatoma cells

The effects of AFP (Zhenglong Biochem.Lab) on cell proliferation were detected using a CCK‐8 kit (Dojindo). Absorbance was measured at 450 nm using a Universal Microplate Reader to analyse cell viability. The cell viability was measured using the following formula: [(A_s_–A_b_)/ (A_c_–A_b_)] × 100%, where As is the sample, Ab the background and Ac the control.

### 2.11 Chromatin immunoprecipitation (ChIP)

The ChIP‐PCR assay was performed in the standard manner to determine the influence of AFP on the capacity of RAR binding to DNA. Specific DNA fragments were obtained, purified and subjected to PCR analysis using a NovoNGS^®^ CUT&Tag High‐Sensitivity Kit (Novoprotein Scientific Inc), following the manufacturer's recommended protocol. Primers used for ChIP‐PCR are listed in Table S1.

### 2.12 Statistical analysis

The results are presented as the mean ± SD from at least three independent experiments. Student's *t* test was used to determine the statistical significance. *P* < .05 was considered to be statistically significant.

## 3 RESULTS

### 3.1 AFP promotes the proliferation and weakens apoptosis of hepatoma cells

**Figure 1:**
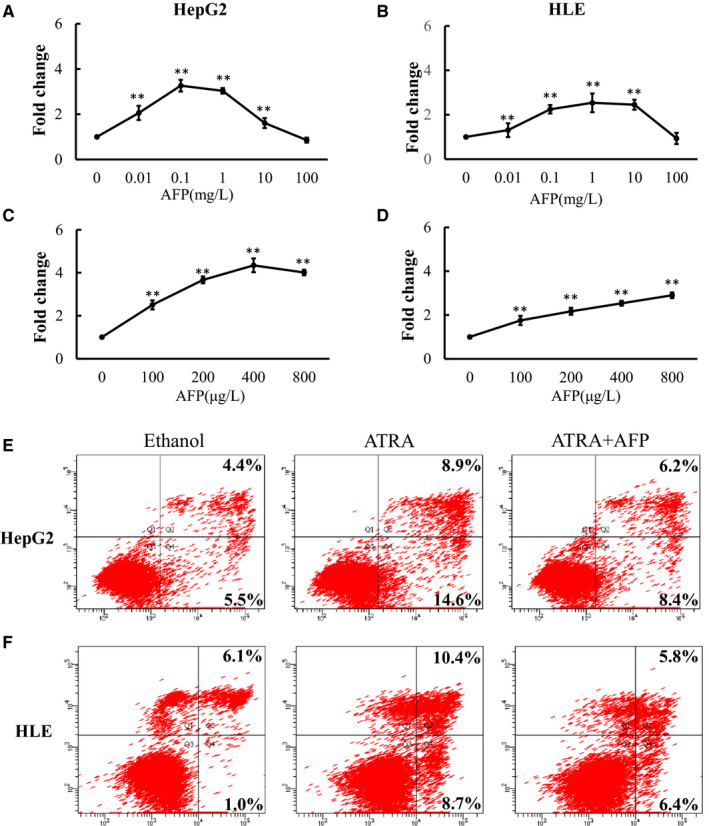
The effect of alpha‐fetoprotein (AFP) on proliferation or apoptosis of hepatoma cells. (A, B) AFP could promote the proliferation of hepatoma cells at the concentration of 0 ~ 100 mg/L. (C, D) AFP has the strongest effect on hepatoma cell proliferation at the concentration of 400 ~ 800 μg/L. (E, F) ATRA promoted apoptosis, whereas AFP protected cells against ATRA‐induced apoptosis in HepG2 and HLE cells. The image is representative of three independent experiments. Data represent mean ± SD of three samples. *P < .05; **P < .01 compared with controls

To investigate the effect of AFP on hepatoma cell proliferation and apoptosis, HepG2 and HLE cells treated with AFP were subjected to CCK‐8 and flow cytometric analyses. Compared with the control group, AFP significantly promoted cell proliferation at different concentrations (HepG2 cells vs control cells: 105.9%, 226.4%, 203.7% and 61.2% when using 0.01, 0.1, 1 and 10 mg/L, respectively; HLE cells vs control cells: 30,7%, 124,6%, 153,7% and 145,5% when using 0.01, 0.1, 1 and 10 mg/L, respectively; all *P* < .05). Among these, AFP concentration of 0.1 ~1 mg/L had the most obvious effect on cell proliferation (Figure 1A,B). Further studies showed that the effect of AFP on the proliferation of HepG2 and HLE cells was most significant at 400 ~ 800 μg/L, which was roughly 4 or 3 times higher than that of the control group (Figure 1C,D). In addition, the effect of AFP on promoting the proliferation of HepG2 cells was more obvious than that of HLE cells, and the proliferation peak appeared at a lower concentration. We speculate that HepG2 cells express more AFP and thus are more sensitive to the proliferation‐promoting effect of AFP.

Furthermore, flow cytometry showed that the apoptotic percentage of hepatoma cells was significantly increased after 36‐hour treatment with ATRA compared with the control group (137.4% and 169.1% in HLE cells and HepG2 cells, respectively vs. control cells; all *P* < .05). As expected, higher apoptosis was observed in HLE cells compared to HepG2 cells (AFP‐positive cells). However, the apoptotic effect of ATRA was reversed after the addition of AFP and compared with the ATRA group (37.9% and 36.1%, in HLE cells and HepG2 cells, respectively, vs control cells; all *P* < .05) (Figure 1E,F). These results suggest that AFP can enhance the anti‐apoptotic ability of cells and help cells to resist the transmission of apoptotic signals.

### 3.2 Cytoplasmic AFP interacts with RAR and interrupts its translocation to the nucleus

**Figure 2:**
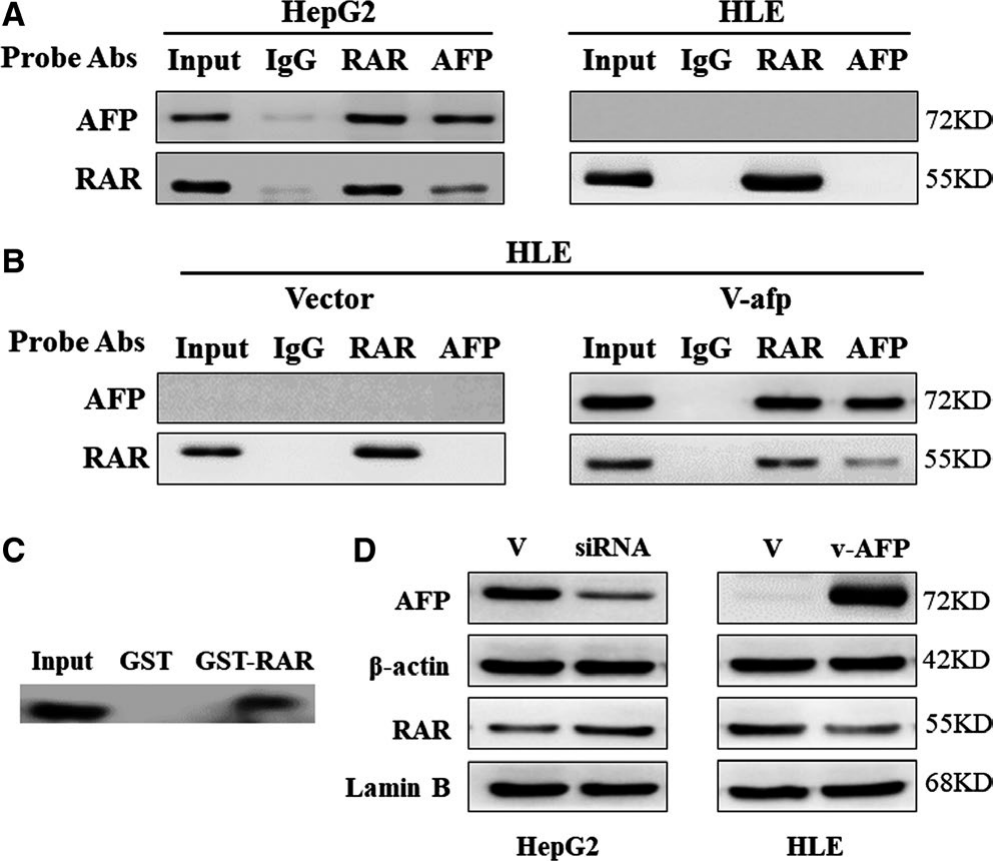
The interaction between AFP and RAR in hepatoma cells. (A) CoIP analysis of the interaction of AFP and RAR in HepG2 and HLE cells. (B) CoIP analysis of the interaction of AFP and RAR in pcDNA3.1‐*afp*‐transfected HLE cells. (C) GST pull‐down analysis of the interaction of AFP and RAR in HepG2 cells. (D) Analysis with Western blotting of effects of AFP on nuclear translocation of RAR in HepG2 and HLE cells. Data are representative of an experiment that was repeated at least three times

To clarify the molecular mechanism through which AFP exerts its action and verify the predicted results of bioinformatics, HepG2 cells and HLE cells were analysed for protein immunocoprecipitation. AFP interacted with RAR in the cytoplasm of HepG2 cells, as demonstrated by CoIP and GST pull‐down analysis. This interaction was undetectable in HLE cells, which lacked AFP. When AFP is expressed in pcDNA3.1‐*afp*‐transfected HLE cells, interaction of AFP and RAR was detectable (Figure 2A‐C). As a transcription factor, RAR needs to enter the nucleus, where it affects the transcription of downstream genes that regulate proliferation, apoptosis and other cell biological behaviours. To further examine the interaction of AFP with RAR, we inhibited AFP in HepG2 cells and overexpressed AFP in HLE cells. As expected, inhibition of AFP in HepG2 cells enabled RAR translocation in the nucleus, whereas transfection of pcDNA3.1‐*afp* into AFP‐negative HLE cells inhibited the translocation (Figure 2D). These interactions suggest that these molecules have high functional significance in complex signalling networks. These results also provide evidence for the role of AFP.

### 3.3 Expression of the Bcl‐2 is inversely correlated with RAR activation

**Figure 3:**
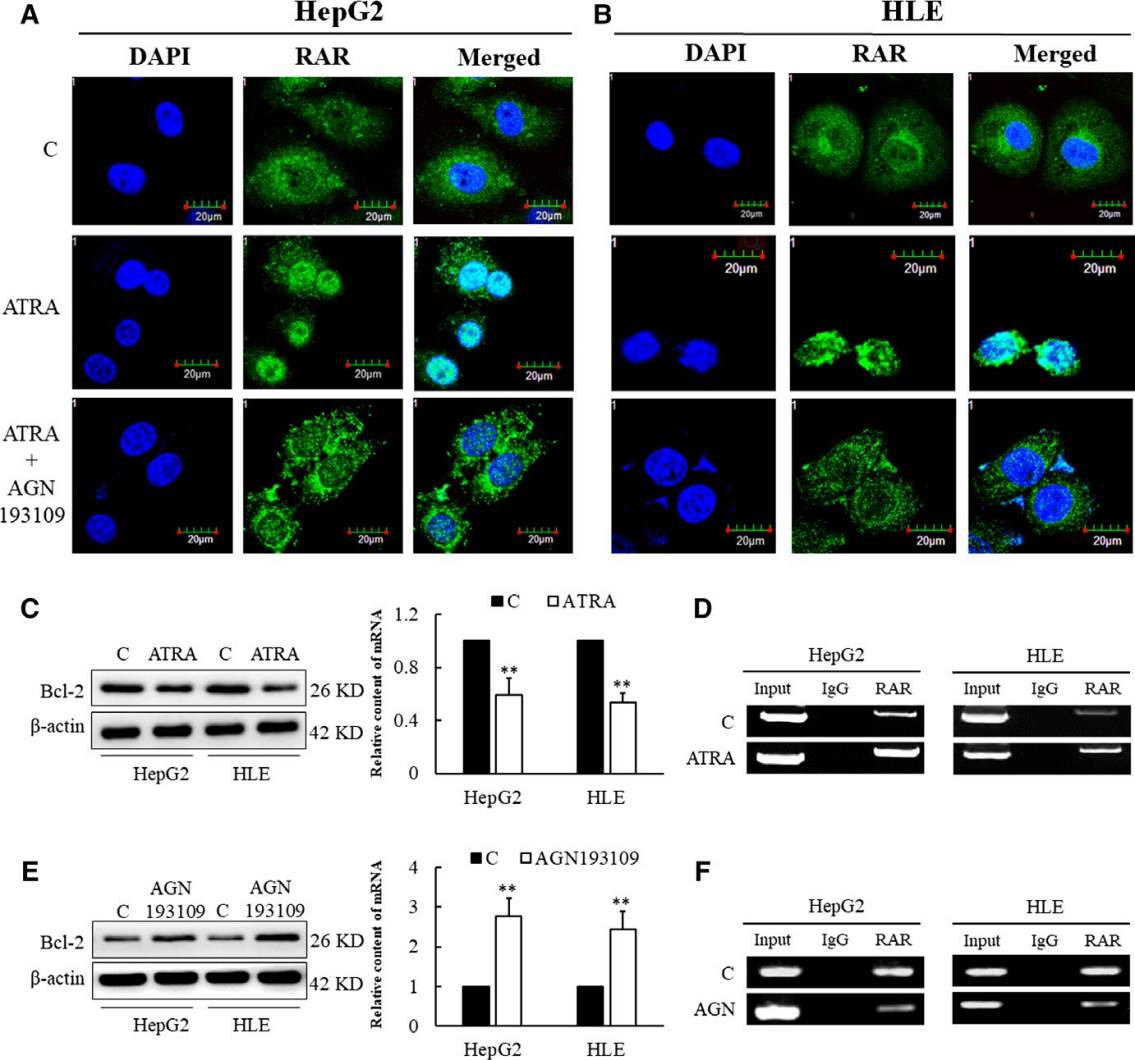
Effect of RAR in expression of Bcl‐2 in HepG2 and HLE cells. (A, B) ATRA induces RAR nuclear translocation, whereas AGN blocks its translocation in HepG2 and HLE cells. Cells were treated with ATRA or ATRA plus AGN193109. Translocation of RAR in HepG2 and HLE cells was viewed and captured by laser confocal microscope. Nuclei and RAR were stained with DAPI (blue) and Alexa Fluor 488 (green), respectively. (C) ATRA inhibits the expression of Bcl‐2 detected by Western blot and qRT‐PCR. (D) Effects of ATRA administration on RAR nuclear entrance analysed by ChIP assay in HepG2 and HLE cells. (E) Retinoic acid receptor antagonist AGN193109 promotes the expression of Bcl‐2 evaluated with Western blot and qRT‐PCR. (F) Effects of AGN193109 on RAR nuclear translocation analysed by ChIP assay in HepG2 and HLE cells. These experiments were repeated at least three times. *P < .05 and**P < .01 compared with controls

The involvement of RAR in Bcl‐2 expression was demonstrated by cells treated with ATRA and AGN193109 assay. ATRA is a natural ligand for RAR, whereas AGN193109 is a retinoic acid analogue, a highly effective and specific antagonist of the retinoic acid receptor (RARs). Observation with confocal microscopy showed that ATRA induces RAR entry in the nucleus in HepG2 and HLE cells, which was apparently reduced after the addition of AGN193109 (Figure 3A,B). Likewise, the expression of Bcl‐2 protein and mRNA in both cell lines was reduced after the addition of ATRA (Figure 3C). RAR‐mediated regulation of Bcl‐2 expression was further confirmed with the ChIP assay, which showed that ATRA administration can promote greater binding of RAR to its element in the negative regulatory region of the *Bcl‐2* gene in HepG2 and HLE cells (Figure 3D). The results of Western blotting and qRT‐PCR (Figure 3E) showed that the expression of Bcl‐2 was increased after the addition of AGN193109. The weakened ability of RAR to bind to hormone reaction element (HRE) in AGN193109‐treated HepG2 and HLE cells was detected by ChIP (Figure 3F). These results confirmed that RAR signalling mediates the expression of Bcl‐2.

### 3.4 Effect of AFP on RAR‐mediated biological effect of Bcl‐2

**Figure 4:**
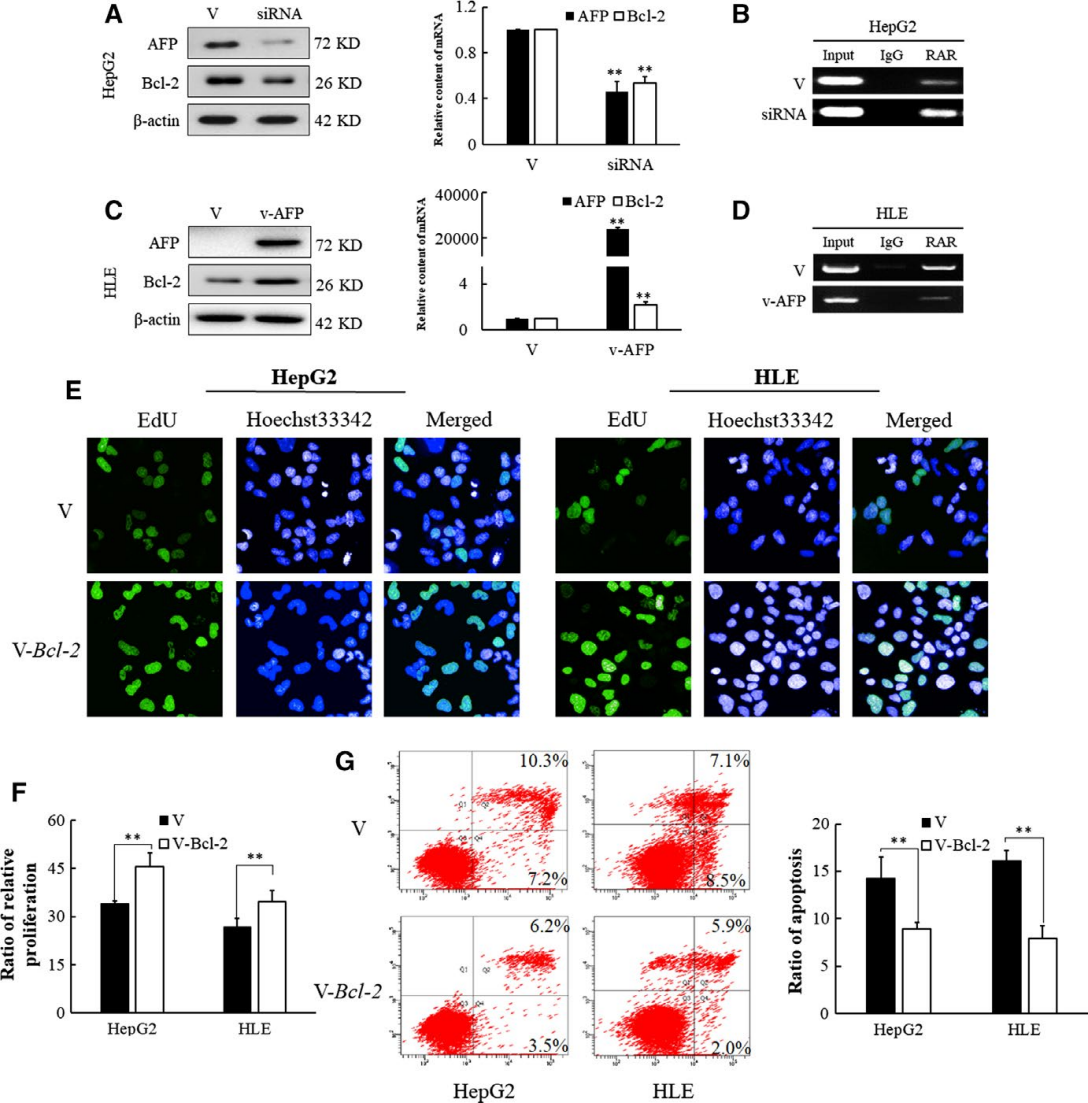
Expression of Bcl‐2 after AFP knockdown/overexpression in HepG2 and HLE cells. (A) Analysis with Western blotting and qRT‐PCR for effects of AFP knockdown on expression of the Bcl‐2 in HepG2. (B) Determination of RAR capacity for binding to DNA in AFP‐silenced HepG2 cells. (C) Analysis with Western blotting and qRT‐PCR for effects of AFP overexpression in expression of the Bcl‐2 in HLE cells. (D) ChIP assays were performed to determine the capacity of RAR for binding to DNA in AFP gene‐transfected HLE cells. Lane 1: input; lane 2: precipitation with non‐specific IgG; lane 3: precipitation with antibody against RAR. (E, F) Bcl‐2 promotes the proliferation in HepG2 and HLE cells. (G) Bcl‐2 inhibits apoptosis induced by ATRA in HepG2 and HLE cells. The image is representative of three independent experiments. *P < .05 and**P < .01 compared with controls

Alpha‐fetoprotein interacted with RAR and, as a consequence, inhibited the nuclear translocation of RAR as described above. The effect of AFP on the RAR signalling pathway might be similar to the effect of antagonist AGN193109. The effect of AFP on Bcl‐2 expression was consistent with the change of AFP. The knockdown of intracellular AFP in HepG2 cells reduced the expression of Bcl‐2 mRNA and protein levels by 40% and 42%, respectively, compared with the control (Figure 4A). The reduction of AFP with siRNA resulted in higher binding of RAR to DNA in HepG2 cells (Figure 4B). In addition, the effect of AFP was also confirmed through the overexpression of the AFP in HLE cells. Bcl‐2 mRNA and protein in HLE cells transfected with pcDNA3.1‐*afp* were significantly increased by up to 2.16‐ and 1.68‐fold as analysed by qRT‐PCR and Western blot, respectively (Figure 4C). Elevated AFP in HLE cells led to an apparent reduction of RAR binding to its elements (Figure 4D).

For further confirmation, the effect of Bcl‐2 on proliferation and apoptosis, a Bcl‐2 expressing plasmid, was constructed and transfected into HepG2 and HLE cell lines. As shown by EdU cell proliferation assays, the pro‐proliferation effect of Bcl‐2 was obvious, and the proliferation rate of HepG2 and HLE cells increased by 29.6% and 34.1%, respectively, compared with the control group (Figure 4E,F). Moreover, the results from flow cytometric analysis showed that overexpression of Bcl‐2 might result in a decrease of the percentage of apoptotic cells induced by ATRA in both of the two hepatoma cell lines (Figure 4G). Compared with group transfected with empty vector, the percentages of apoptotic cells were reduced by 44.6% and 49.4% after transfection with Bcl‐2‐expressing construct, respectively, in which the survival cells were obviously increased.

### 3.5 Alpha‐fetoprotein interacts with RAR and up‐regulates Bcl‐2 in HCC clinical specimens

**Figure 5:**
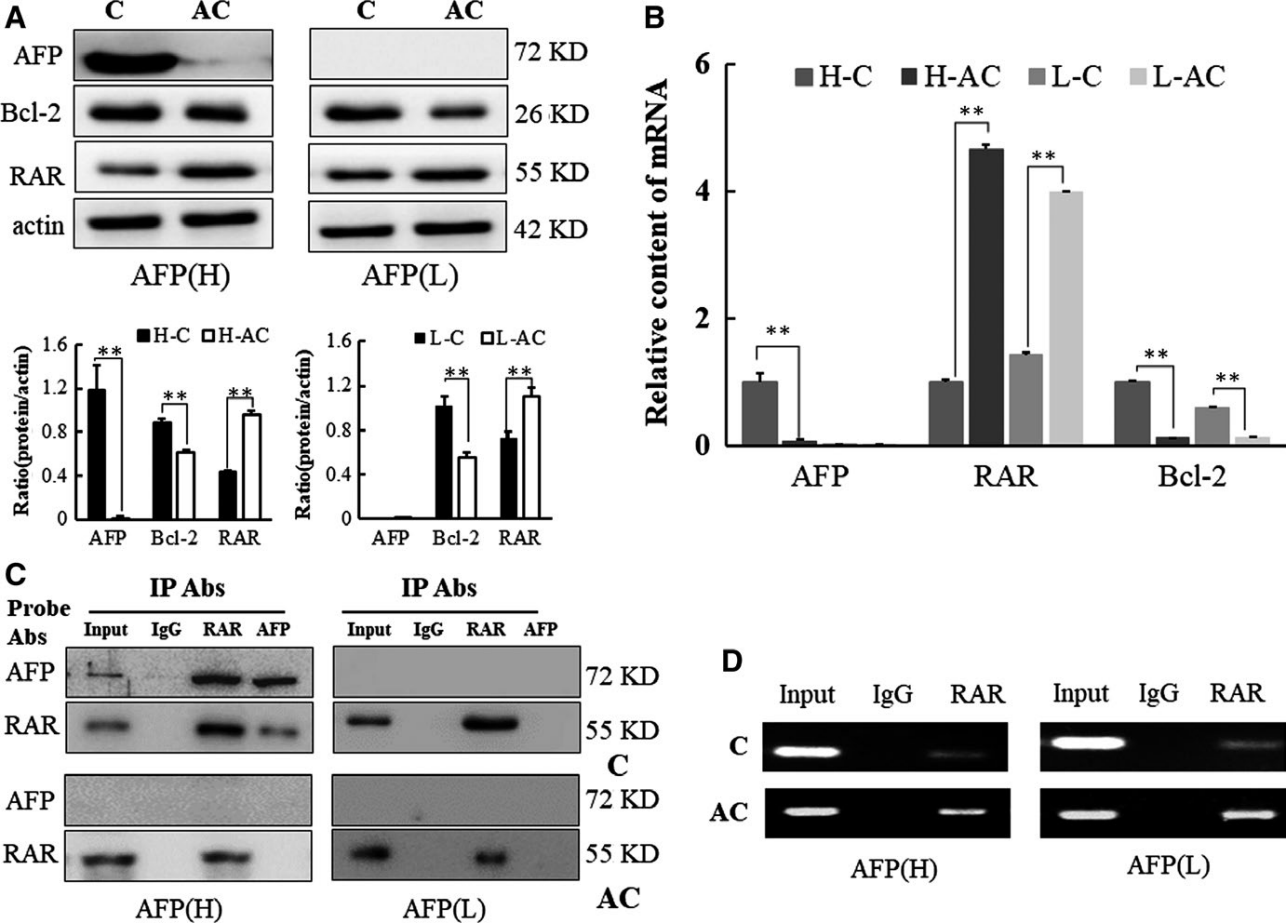
Regulation of RAR in Bcl‐2 expression in clinical HCC specimens. Western blot (A) and qRT‐PCR analysis (B) of expression of AFP, RAR, Bcl‐2 in cancerous and adjacent non‐cancerous tissues. C: cancerous tissues; AC: adjacent non‐cancerous tissues. (C) CoIP analysis of the interaction of AFP and RAR. (D) ChIP analysis of RAR binding to its element in the regulatory region of the Bcl‐2 gene. *P < .05 and**P < .01 as compared with control. These experiments were repeated at least three times

In order to confirm that the interaction between AFP and RAR and that the regulation of RAR on Bcl‐2 expression are universal, a similar experiment was performed on clinical tissue specimens. Western blot (Figure 5A) and qRT‐PCR (Figure 5B) results showed that AFP was detected in tumour tissues with high AFP serum, but not in adjacent non‐cancerous liver tissues, or HCC tissues with low serum AFP levels. It is noteworthy that the significant decrease in the RAR protein level was associated with the up‐regulation of Bcl‐2 protein in the cancer tissues of patients. In addition, the level of Bcl‐2 in cancer tissues of patients with low AFP serum was lower than that of patients with high serum AFP. AFP interaction with RAR was observed in tumour tissues with high serum AFP, but not in adjacent non‐cancerous liver tissues or tumour tissues with low serum AFP (Figure 5C). RAR binds to the negative regulatory region of the Bcl‐2 gene in tissues with high or low serum AFP. However, in view of the low RAR protein showed in the ChIP assay, binding is significantly reduced in the cancer tissues, which suggests that the interaction between elevated AFP in cytoplasm and RAR results in the reduction of RAR translocation to the nucleus (Figure 5D).

## 4 DISCUSSION

Hepatocellular carcinoma is one of the most common malignant tumours in the world, with an increasing incidence. About 600‐700 000 people die of HCC every year, which ranks HCC as the third most common cause of cancer death worldwide. Since the 1970s, AFP has been recognized as a tumour marker for the diagnosis of HCC. Yet, its exact mechanism of action still remains unclear. Previous studies have found that the serum AFP level is positively correlated with the malignant degree of HCC.^8^, ^9^ Patients with high serum AFP usually have large tumours, multiple diffuse tumours, portal vein thrombosis, enhanced infiltration and increased metastasis. Moreover, other studies have found that AFP regulates cell differentiation, growth regulation, tumorigenesis and other processes.^2^, ^20^, ^21^, ^22^ The secreted AFP can affect other adjacent cells by binding to their receptors, thus stimulating tumour cell biological behaviour.^23^, ^24^ So far, studies on the biological significance of AFP have focused on the effect of circulating AFP on tumour development. However, ongoing research is beginning to shed light on the role of cytoplasmic AFP. There is growing evidence that intracellular AFP is involved in tumour growth.^25^, ^26^ Studies have found that cytoplasmic AFP can combine with PTEN to promote the onward transmission signalling of the PI3K/AKT signalling, which leads to aberrant growth of hepatocellular carcinoma cells.^27^ In addition, AFP can form complexes with caspase‐3 but not with caspase‐8, blocking caspase‐cascade signalling, demonstrating the selectivity of AFP in interfering with the apoptotic signalling pathway.^28^ Furthermore, silencing the AFP gene can reduce the expression of mutant p53, resulting in the decrease of Bcl‐2 expression and an increase of Bax expression. Up‐regulation of Bax/Bcl‐2 ratio further triggers the release of cytochrome c, which activates caspase‐3 to induce apoptosis, suggesting that AFP inhibits apoptosis through the p53/Bax/caspase‐3 apoptotic signalling pathway.^29^ Based on the prediction that cytoplasmic AFP could bind to a variety of receptors, including retinoic acid receptors,^12^ and the previous studies that AFP can interfere with signalling pathways and silencing the AFP gene can reduce the expression of Bcl‐2, our study confirmed that AFP is one of the main binding partners of RAR and interferes with the RA‐RAR signalling pathway to promote Bcl‐2 expression.

**Figure 6:**
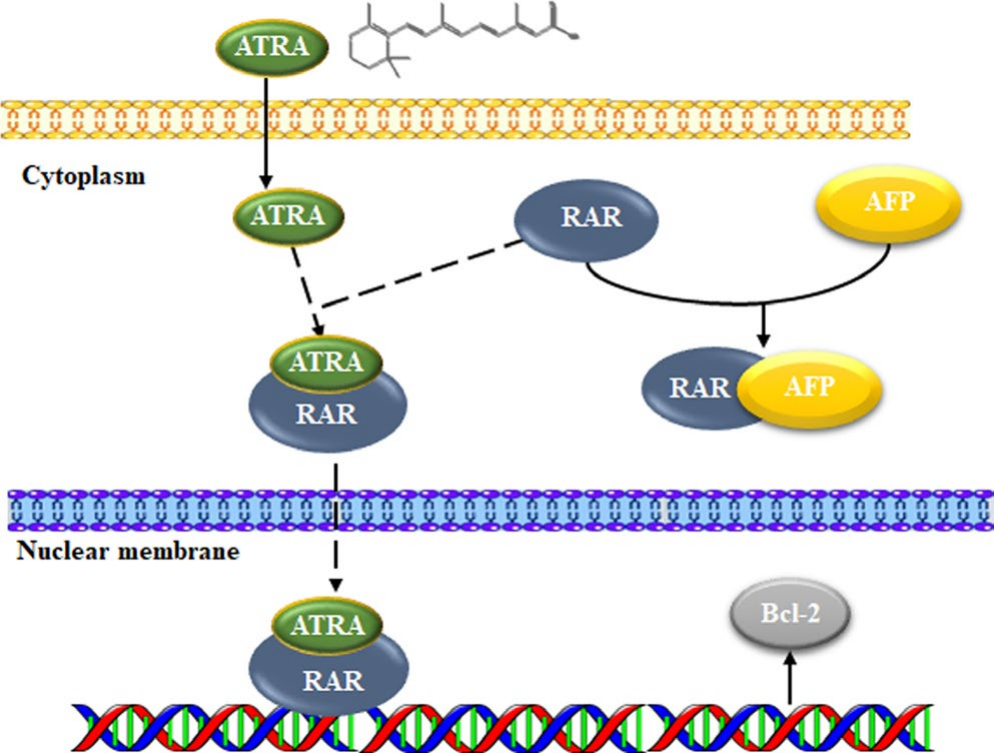
Schematic model of the possible role of cytoplasmic AFP in RAR‐mediated expression of Bcl‐2

Our study confirmed that AFP can promote the proliferation of HCC cells and protect HCC cells against ATRA‐induced apoptosis (Figure 1). Furthermore, it was further discovered that AFP could interact with nuclear transcription factor RAR, interfering an RA‐RAR signalling pathway and thereby inhibiting the transcriptional regulatory role of RAR in the cell (Figure 2). ATRA, the natural ligand of RAR, and AGN193109 (RARs antagonist) can stimulate or antagonize RAR activation, respectively, and subsequently inhibit or promote the expression of Bcl‐2 (Figure 3). ATRA has been used as a first‐line chemotherapy agent in the treatment of leukaemia because of its role in inducing malignant cell differentiation. However, human hepatoma cells are resistant to the apoptotic effect of ATRA and are prone to drug resistance.^30^ Our results indicated that AFP binds to RAR, which competitively reduces the chance of ATRA binding to RAR (Figure 6). Meanwhile, we also found that the overexpression of AFP in HLE cells was positively correlated with the elevated expression level of Bcl‐2 and that the inhibition of AFP in HepG2 cells could constrain the expression level of Bcl‐2 (Figure 4). Earlier studies found that the negative regulatory region of the 5'‐untranslated region of the *Bcl‐2* gene contained RAR binding sites, which inhibited the activity of the promoter.^31^, ^32^ Thus, the Bcl‐2 promoter could be deactivated by the retinoic acid receptor.^17^, ^33^ Our data suggested that the interaction of AFP and RAR could inhibit the activation of RAR, thus promoting the expression of Bcl‐2, which is consistent with the effect of using retinoic acid receptor antagonist AGN193109. These data were consistent with previous studies.^17^ Bcl‐2 is a well‐known pro‐carcinogenic factor. In this study, we overexpressed Bcl‐2 in hepatoma cells and then treated those cells with ATRA. We found that the apoptosis rate in those cells was significantly reduced compared to control cells, which further confirmed the anti‐apoptotic effect of Bcl‐2 (Figure 4).

**Figure 7:**
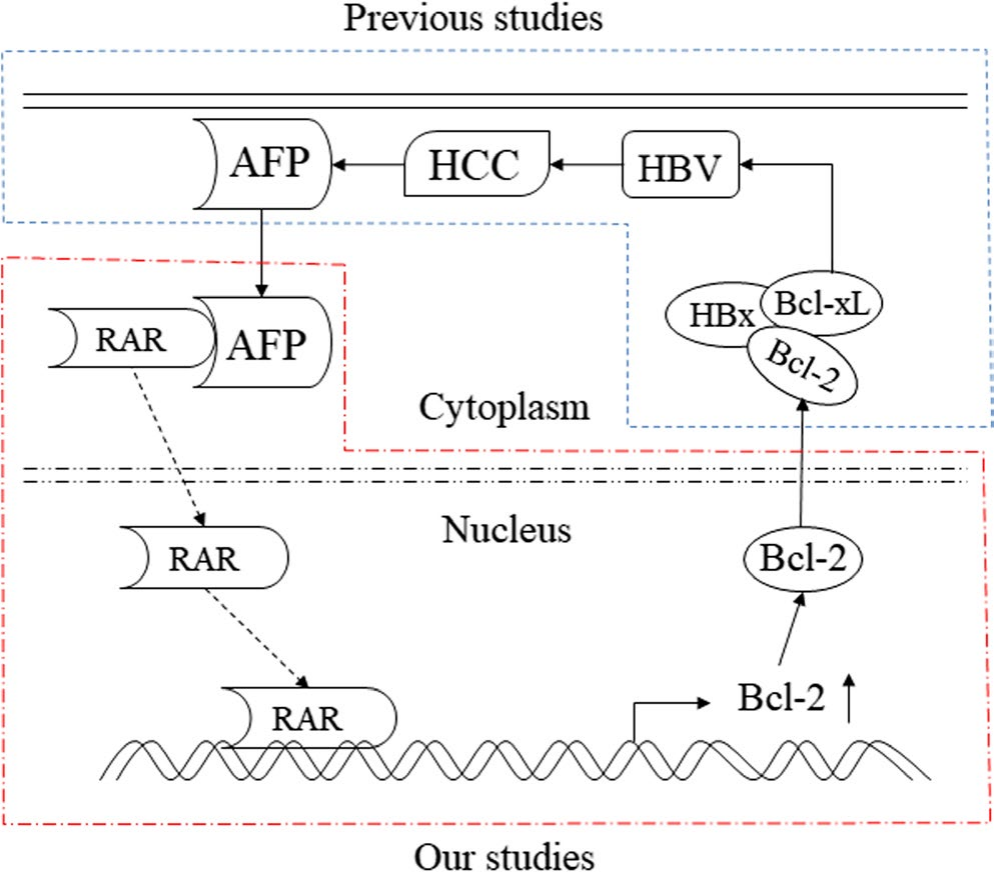
Schematic model of the relationship between HBV, HCC, AFP and Bcl‐2

Approximately 2 billion people are infected with the hepatitis B virus worldwide, and more than 350 million people are chronic HBV carriers.^34^ Recent estimates attribute over 50% of HCC cases worldwide to HBV, making it the most common carcinogenic factor for HCC.^35^, ^36^ Besides, 70%‐80% of HCC patients are accompanied by abnormal expression of AFP.^37^ Meanwhile, HBx was found to interact with the anti‐apoptotic proteins Bcl‐2 and Bcl‐xL through a Bcl‐2 homology 3 (BH3)‐like motif and subsequent elevate cytosolic calcium, which is required for HBV viral replication in mammalian cells.^38^ Summarizing the above studies, we found that the main aetiology of HCC was HBV, whose replication was related to Bcl‐2, and the frequently abnormal expression of AFP in HCC could promote the expression of Bcl‐2. HBx targets Bcl‐2 proteins to promote viral replication during HBV pathogenesis. Our work complements the chain of evidence that make HBV, HCC, AFP and Bcl‐2 a cohesive whole (Figure 7). Our study presents an excellent therapeutic intervention point for the treatment of HCC patients, as well as provides a supplementary explanation of the pathogenesis of HCC, especially HBV‐associated HCC.

## 5 CONCLUSION

In summary, AFP interferes with the cascade conduction of the RA‐RAR signalling pathway by regulating the transcription factor RAR, thus reducing the sensitivity of hepatoma cells to the chemotherapy drug ATRA. In addition, AFP promotes the expression of Bcl‐2. Therefore, our research results could be used as support for new ideas and strategies for the treatment of HCC. When treating patients with AFP‐positive HCC, AFP can be used as a target molecule. If we silence or inhibit the expression of the AFP gene, it may enhance the inhibition of the process of HCC and enhance the therapeutic effect of HCC. Further studies should examine whether the interaction between AFP and RAR can regulate other genes related to proliferation/apoptosis.

## CONFLICT OF INTERESTS

The authors declare no competing interests.

## AUTHOR CONTRIBUTIONS

**Chao Zhang:** Conceptualization (lead); Data curation (lead); Methodology (lead); Project administration (lead); Writing‐original draft (lead). **Jiangtao Zhang:** Writing‐review & editing (supporting). **Jing Wang:** Writing‐review & editing (supporting). **Ying Yan:** Writing‐review & editing (supporting). **Chuanbao Zhang:** Conceptualization (supporting); Project administration (equal); Writing‐review & editing (supporting).

## ETHICS APPROVAL AND CONSENT TO PARTICIPATE

The use of human tissues was approved by the Ethical Committee of Beijing Hospital and conforms to the Helsinki Declaration and to local legislation.

## Supporting information

Table S1
