# Supplementary material for: Alpha‐fetoprotein accelerates the progression of hepatocellular carcinoma by promoting Bcl‐2 gene expression through an RA‐RAR signalling pathway
Source: J Cell Mol Med. 2020 Oct 22;24(23):13804–12. doi: 10.1111/jcmm.15962 (PMC7753843; doi:10.1111/jcmm.15962)
Supplement: Supplementary file 1 — Table S1 [file JCMM-24-13804-s001.docx]

Supplementary Table 1. Sequences of oligonucleotides used for qRT-PCR, ChIP-PCR, plasmid constructs assays.

| Oligonucleotides sequences Product size(bp) | | | |
| --- | --- | --- | --- |
| **Primers for qRT-PCR** | | | |
| AFP | Sense | 5'-CCAACAggAggCCATgCTT-3' | 61 |
|  | Antisense | 5'-gAATgCAggAgggACATATgTTT-3' |  |
| Bcl-2 | Sense | 5'-CTGGGATGCCTTTGTGGAACT-3' | 65 |
|  | Antisense | 5'-ACAGCCAGGAGAAATCAAACAGA-3' |  |
| RAR | Sense | 5'-GGCTACCAAGTGCATCATCAA-3' | 133 |
|  | Antisense | 5'-TGTGCAGATACGCAGCATCAG-3' |  |
| β-actin | Sense | 5'-ccaaccgcgagaagatga-3' | 72 |
|  | Antisense | 5'-ccagaggcgtacagggatag-3' |  |
| **Primers for PCR** | |  |  |
| Bcl-2 | Sense | 5'-CGTCCTGCCTTCATTTATCCAG-3' | 460 |
|  | Antisense | 5'-ATCCCATCAATCTTCAGCACTC-3' |  |
| **Primers for plasmid constructs** | |  |  |
| Bcl-2 | Sense | 5'-CAAGCTTatggcgcacgctgggagaa-3' |  |
|  | Antisense | 5'-GGAATTCTCACTTGTGGCCCAGATAGG-3' |  |
